# Effect of needle puncture and electro-acupuncture on mucociliary clearance in anesthetized quails

**DOI:** 10.1186/1472-6882-6-4

**Published:** 2006-02-23

**Authors:** Shusheng Tai, Jiulin Wang, Feng Sun, Stevenson Xutian, Tianshan Wang, Malcolm King

**Affiliations:** 1 Pulmonary Research Group, 173 Heritage Medical Research Center, University of Alberta, Edmonton, AB T6G 2S2 Canada; 2 Acupuncture Program, Grant MacEwan College, 10700-104 Avenue, Edmonton AB T5J 4S2 Canada

## Abstract

**Background:**

Acupuncture therapy for obstructive respiratory diseases has been effectively used in clinical practice and the acupuncture points or acupoints of Zhongfu and Tiantu are commonly-used acupoints to treat patients with the diseases. Since the impaired mucociliary clearance is among the most important features of airway inflammation in most obstructive respiratory diseases, the effect of needle puncture and electro-acupuncture at the specific acupoints on tracheal mucociliary clearance was investigated in anesthetized quails.

**Methods:**

Mucociliary transport velocity on tracheal mucosa was measured through observing the optimal pathway, and fucose and protein contents in tracheal lavages were determined with biochemical methods. In the therapeutic group, needle puncture or electro-acupuncture stimulation to the acupoints was applied without or with constant current output in 2 mA and at frequency of 100 Hz for 60 minutes. In the sham group, electro-acupuncture stimulation to Liangmen was applied.

**Results:**

Our present experiments demonstrated that the electro-acupuncture stimulation to Zhongfu and Tiantu significantly increased tracheal mucociliary transport velocity and decreased the content of protein in the tracheal lavage, compared with the control group. Moreover, either needle puncture or electro-acupuncture stimulation to Zhongfu and Tiantu significantly reverted the human neutrophil elastase-induced decrease in tracheal mucociliary transport velocity and human neutrophil elastase -induced increase in the contents of fucose and protein in the tracheal lavage, compared with the control group.

**Conclusion:**

These results suggest that either needle puncture or electro-acupuncture stimulation to the effective acupoints significantly improves both airway mucociliary clearance and the airway surface liquid and that the improvements maybe ascribed to both the special function of the points and the substantial stimulation of electricity.

## Background

Long ago, Chinese people observed that stimulation to different points of human body could produce different sensations (sour, numbness, swelling, pain, etc.), physiological reactions (laughing, spontaneous limb movement, etc.) and therapeutic effects (alleviation of toothache, relief of dyspnea, etc.). Since these ancient times, the stimulations of heating with a lighted moxa (moxibustion), pressure with a human finger (acupressure), cupping with a vacuumed cup (cupping) and needling with a sterilized needle (acupuncture) have been extensively applied by Chinese clinicians to treat different diseases. With long-term practice from generation to generation, clinicians recognized that the points with same or similar effects were regularly located in a line. For example, the points in the line from the chest to the hand were effective for diseases in lung, trachea, pharynges and other body parts mostly related to respiratory system. This line was then termed the lung meridian, where the points are called lung meridian acupuncture points (acupoints) [1,2].

The lung meridian acupoints have been accurately described in anatomic knowledge and located with scientific measurements, and their effects and manipulation methodologies have been introduced in detail. For example, Zhongfu ("Central Treasury"), as the first acupoint of Lung meridian, is named LU1. It is laterosuperior to the sternum at the lateral side of the first intercostals space, at 3/4 of distance from the frontal midline to the peak of shoulder. It alleviates cough, dyspnea, pain in the chest, shoulder and back, fullness of the chest, etc. Tiantu ("Heavenly Chimney"), as the 22nd acupoint of the Conception vessel, is named CV22. It is located at the center of the suprasternal fossa. It alleviates dyspnea, cough, sore throat, dry throat, hiccup, sudden hoarseness of the voice, difficulty in swallowing, goiter, etc. Liangmen ("Beam Gate"), as the 21st acupoint of the stomach meridian, is named ST21. It is located at 1/3 of the distance from the frontal midline to the edge of the body and at the level of 1/4 of distance from sternocostal angle to anus. It is used to treat gastric pain, vomiting, anorexia, abdominal distension, diarrhea, etc. [1,2].

Acupuncture therapy for obstructive respiratory diseases has been effectively used in clinical practice in most Oriental countries for thousands years [3,4]. In more recent times, this form of acupuncture therapy has attracted considerable attention in treating obstructive respiratory diseases (asthma, rhinosinusitis, chronic obstructive pulmonary diseases (COPD)) in Western countries [5-9]. The National Institute of Health (NIH) in a consensus statement derived from a Fall of 1997 consensus conference to assess acupuncture has indicated that acupuncture was useful in pain control and maybe a useful adjunct treatment for the management of asthma [10]. Since many of the issues surrounding the clinical use of acupuncture relate to a perceived lack of scientific evidence, an evidence-based experimental approach should help to address some of these concerns.

Impaired mucociliary clearance (MC) mainly resulting from viscous airway secretion, resulting in airway obstruction, is not only among the most important features of airway inflammation in most obstructive respiratory diseases (e.g. COPD, asthma, acute bronchitis, cystic fibrosis, bronchiectasis) but also the major cause of death or poor quality of life in patients with the above respiratory diseases [11].

Since the needling manipulation of acupuncture is a special technique, only well-trained clinicians are authorized as acupuncture practitioners or acupuncturists. The stimulation by different acupuncturists may vary greatly, so the manipulation of needling is sometimes performed by electro-stimulation or electro-acupuncture (EA), in which the stimulation intensity can be regulated accurately by the current output and frequency, especially for acupuncture experiments. In recent years, electro-acupuncture has been employed by more and more acupuncturists worldwide to treat the above diseases in the clinic [12].

Although either regular acupuncture or electro-acupuncture has been demonstrated to facilitate nasal mucociliary clearance in patients with chronic rhinitis and polypous rhinosinusitis [13,14], the research papers on the efficacies and mechanisms in animal models are limited. To investigate the efficacies and mechanisms, we applied both needle puncture (NP) and EA to our well-developed quail model, where human neutrophil elastase (HNE) was applied to simulate the human airway inflammatory conditions with impaired MC [15].

The contents of viscous components in the airway secretion, which are the key factors to airway secretion viscoelasticity (the key rheological property of airway secretion to affect MC), are thought to play an important role in impaired MC during inflammation status. We hypothesized that NP or EA may facilitate MC and that the facilitation of MC by NP or EA may partly result from decreasing the contents of viscous mucin and other macromolecules in airway secretion.

To confirm the above hypotheses, mucociliary transport velocity (MTV, a directly measurable parameter of MC) was measured, and fucose (a saccharide marker of viscous epithelial mucins) and protein (representative of macromolecules) contents were determined in the present experiment.

## Methods

Male quails, weighing 100–120 g, were obtained from Laboratory Animal Services of the University of Alberta (Edmonton, Alberta, Canada). The following study design was approved by Health Sciences Animal Policy and Welfare Committee of University of Alberta. The birds were intraperitoneally anesthetized with sodium pentobarbital at dose of 15 mg/kg body weight [15,16]; supplemental sodium pentobarbital was administered as necessary. The anesthetized quail was immobilized on its back and the neck feathers were removed. The neck skin was incised along the midfrontal line and the wound margins were clipped with two celfins to keep the incision slightly open. The local blood vessels and connective tissues were carefully dissected to expose the trachea.

A suture was passed under the exposed trachea so that it could be elevated to facilitate the operation. An aperture approximately 1 × 2 mm in size was made in the trachea with a heated surgical blade. As soon as the operation was complete, the acupuncture needles (0.20 mm diameter, 13 mm length, supplied by University Acupuncture and Herbal Therapy Center, Edmonton, Alberta, Canada) were inserted into the quail and the quail was then placed in an observation chamber. There, its trachea was ventilated with warmed, moistened air (about 38°C and approximately 100% relative humidity) by the use of a humidifier (Cascade 1900, Bennett Respiration Products, Los Angeles, California, USA). A thermometer was placed in the chamber to monitor the temperature.

In the observation chamber, powdered paper ash 10–20 μm in diameter, made in our laboratory, was placed on the caudal section of the tracheal mucosa of the quail with a fine plastic stick to determine the pathway for optimal MTV. MTV was thereafter measured at the same site, using a microscope, on the basis of the time taken for the powder to move 1 mm on the mucosa. MTV measurements were started 30 min after the trachea was exposed. The MTV was calculated by dividing the distance traveled (mm) by the time elapsed (seconds); at least one measurement was made per minute to minimize measurement variability.

HNE (elastase, from human sputum, 875 units/mg protein) was obtained from Elastin Products Inc. (Owensville, Missouri, USA). All other reagents were purchased from Fisher Scientific (Nepean, Ontario, Canada). The HNE stocks were dissolved in phosphate buffered saline without Ca^2+ ^and Mg^2+ ^(designated as PBS (-)) and prepared into appropriate solution concentrations. In the observation chamber, a 2-μl aliquot of either HNE solution or PBS (-) solution was applied directly onto the tracheal mucosa. Either HNE or PBS (-) solution was applied 45 min after the trachea was exposed.

EA stimulation was administered via stainless steel needles. 3–5 mm of the needle length was inserted into selected acupoints. The exposed parts of the needles were bent so that they were easily fixed in place. To avoid possible electrical leakage, the needles were covered by plastic tubes.

LU1 and CV22 were selected as effective acupoints in therapeutic groups, while ST21 was selected as sham acupoint in non-effective group. The needles were connected to an electro-stimulator (ITO IC4107 Seard, Electro-Therapeutic Devices Inc., Markham, Ontario, Canada), which delivers square wave pulse of 0.3 ms pulse width, with constant current output in 2 mA. The frequency was set in 100 Hz. EA stimulation was set for 60 minutes, starting 15 minutes after MTV measurement. NP stimulation was performed on the same manner as in EA stimulation group except without turning on the electrical stimulator. In control animals, no needles were inserted.

The MTV was measured for 75 min. The MTV results were calculated by the following equation: V_t_/V_0 _× 100 %, where V_t _is the average velocity in each 5 min interval and V_0 _the average velocity in the last 5 min interval before application of the test solution.

To know whether NP or EA affects the components of airway secretion, tracheal lavages were collected and analyzed on the following manner [15]. In the observation chamber, HNE, PBS (-) solution, NP or EA stimulation was applied in other quails 45 min after the quail trachea was exposed as described above. The quail was removed from the observation box 25 min after the NP or EA stimulation started. The suture was knotted tightly around the trachea 10 mm below the aperture and the portion of the trachea between the knot and the aperture was severed and removed. The airway secretion in the tracheal segment was collected by tracheal lavage with 200 μl of PBS (-) solution. Insoluble materials were removed from the tracheal lavage sample by centrifugation (12,000 × g, 1 min). The supernatant was stored at -30°C for fucose and protein analyses. The fucose content was quantitated by means of the Gibbons method and the protein was assayed using the Bradford method [17,18].

All the values were expressed and presented as the means ± S.E. (n = 5). Statistical analyses were performed using Fisher PLSD (ANOVA) and a P value < 0.05 was considered significant.

## Results

**Figure 1:**
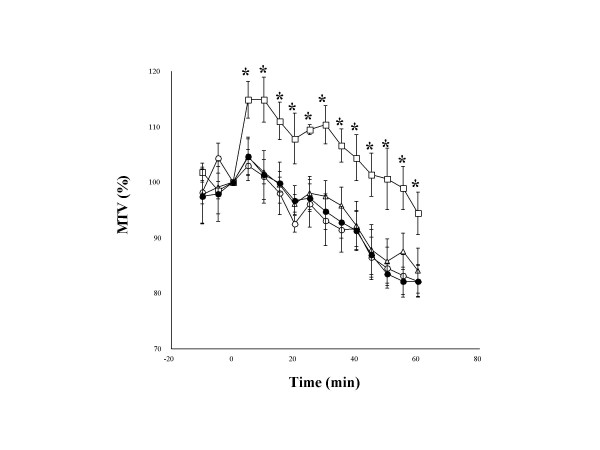
**The effect of needle puncture (NP) and electro-acupuncture (EA) on mucociliary transport velocity (MTV) in anesthetized quails**. Values are expressed as percentages of the respective pre-application values for five animals. Each point and vertical bar represent the mean value and S.E. The electro-stimulator delivers square wave pulse of 0.3 ms pulse width, with constant current output in 2 mA. The frequency is set in 100 Hz. EA stimulation is set for 60 minutes, starting 15 minutes after MTV measurement. ○: Control, △: NP to Zhongfu and Tiantu, □: EA to Zhongfu and Tiantu (EA_1_), ●: EA to Liangmen (EA_2_). *: P < 0.05, statistically significant difference from the control value (ANOVA).

**Figure 2:**
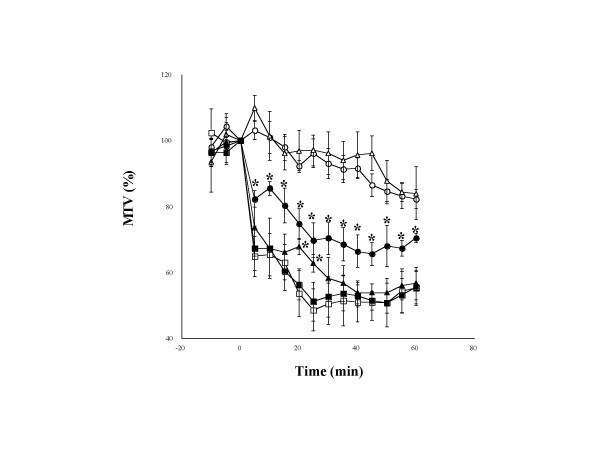
**The effect of needle puncture (NP) and electro-acupuncture (EA) on human neutrophil elastase (HNE)-induced decrease in mucociliary transport velocity (MTV) in anesthetized quails**. Values are expressed as percentages of the respective pre-application values for five animals. Each point and vertical bar represent the mean value and S.E. The electro-stimulator delivers square wave pulse of 0.3 ms pulse width, with constant current output in 2 mA. The frequency is set in 100 Hz. EA stimulation is set for 60 minutes, starting 15 minutes after MTV measurement. ○: Control, △: PBS(-), □: 300 μg/kg of HNE, ●: 300 μg/kg of HNE and EA to Zhongfu and Tiantu (EA_1_), ▲: 300 μg/kg of HNE and NP to Zhongfu and Tiantu, ■: 300 μg/kg of HNE and EA to Liangmen (EA_2_). *: P < 0.05; statistically significant difference from the group given 300 μg/kg of HNE (ANOVA).

Our present experiments demonstrated that the EA stimulation to LU1 and CV22 significantly increased MTV, whereas either EA stimulation to ST21 or NP stimulation to LU1 and CV22 failed to do so (Figure 1). Application of 300 μg/kg of HNE significantly decreased MTV for a period of at least one hour. This HNE-induced decrease in MTV was markedly attenuated by either EA or NP stimulation to LU1 and CV22 but not by EA to ST21 (Figure 2).

EA to LU1 and CV22 was found to significantly decrease protein content but not fucose content, whereas not only NP to LU1 and CV22 but also EA to ST21 failed to change either of these contents. The fucose and protein contents in the tracheal lavages from the HNE (300 μg/kg)-treated group were significantly higher than those of the PBS(-) group. Moreover, the HNE-induced increases in fucose and protein contents were significantly restored by both EA and NP to LU1 and CV22 but not by EA to ST21 (Table 1).

**Table 1:** The effect of needle puncture (NP) and electro-acupuncture (EA) on fucose and protein contents. The tracheal lavages from anaesthetized quails were collected 25 min after the human neutrophil elastase (HNE) or EA treatment. The fucose and protein contents are expressed as μg/10 mm of the tracheal segment (TS). Each value represents the mean value and S.E. for five animals. NP indicates NP to Zhongfu and Tiantu, EA_1 _indicates EA to Zhongfu and Tiantu, EA_2 _indicates EA to Liangmen. *: P < 0.05 vs. control group. #: P < 0.05 vs. the group of HNE (ANOVA).

**Group**	**Fucose (μg/TS)**	**Protein (μg/TS)**
Control	2.13 ± 0.43	18.25 ± 2.17
PBS(-)	2.25 ± 0.39	17.38 ± 3.57
NP	2.17 ± 0.31	16.07 ± 2.61
EA_1_	2.07 ± 0.28	9.18 ± 1.38*
EA_2_	2.21 ± 0.33	19.48 ± 3.78
HNE	8.63 ± 0.71*	55.42 ± 4.13*
HNE+NP	7.17 ± 0.36#	46.03 ± 3.04#
HNE+EA_1_	5.31 ± 0.45#	40.69 ± 1.58#
HNE+EA_2_	8.27 ± 0.50	52.67 ± 3.65

## Discussion

Impaired MC resulting from abnormal and excessive viscous airway secretion is among the most important features of airway inflammation in obstructive respiratory diseases. It is well known that HNE, as a major inflammatory mediator, plays a very important role in the development of MC impairment during the inflammation.

In our previous experiments in a quail model for the study of MC and airway secretion in the trachea, we demonstrated that HNE causes slowing of tracheal MC, and leads to an increase of the contents of airway viscous secretion. Importantly, HNE produces a long-lasting disorder, which lasts for more than one hour. This is sufficient time to apply and monitor the effects of potential therapies. In this model, we have found that ONO-5046 (an elastase inhibitor), Maimendongtang (a classic Chinese herbal prescription), *Radix Ophiopogonis *(RO, the major herb of Maimendongtang) and ophiopogonin-D (an active component of RO) significantly inhibited either the HNE-induced decrease in MTV in tracheal mucosa or the increases in the contents of focuse and protein contents in tracheal lavages [19-21]. In our present studies using the newly-modified quail model, we also demonstrated that HNE caused a reduction in MTV for at least one hour, which agrees with our previous results achieved in the original quail model. Moreover, we found that EA or NP manifested very similar mucoactive effects to those exhibited by the above drugs.

In the non HNE-treated quail model, EA to LU1 and CV22 caused a significant increase in MTV, which might be partly related to its decreasing airway secretion viscoelasticity because the protein content in tracheal lavage was decreased. However, either NP to LU1 and CV22 or EA to ST21 manifested no significant effects on MTV and the components. These results suggested that the selection of effective acupoints is an essential factor and electric stimulation is an important factor.

In the HNE-treated quail model, either EA or NP to LU1 and CV22 inhibited the decrease in MTV and the increases in both protein and fucose contents of tracheal lavages. However, EA to ST21 had little effect on MTV and the components. Since HNE has been demonstrated to cause hypersecretion of mucin, mucin might be a main source of the accumulated protein and fucose in the tracheal lavages [22]. Taken together, it is probable that NP or EA may inhibit the HNE-induced decrease in MTV partly through restoring HNE-induced increase in the contents of protein and fucose, and that EA or NP may restore the HNE-induced increase in the contents through inhibiting the HNE-induced hypersecretion of mucin. On the other hand, it can not be totally excluded that the subsequent decrease in protein and fucose in tracheal lavages may be partly attributed to the facilitation of MTV.

In this experiment, a successful animal pathogenic model was developed, but the effects of NP or EA on the model was observed for only one hour. It is an acute model. Since there are both acute and chronic obstructive respiratory diseases, further experiments on a chronic model are needed to observe a long-term effect. In addition, NP or EA was found to facilitate MTV and decrease the contents of fucose and protein in airway. The primary mechanism may be that the facilitation of TMV partly results from the decrease of the mucin secreted from the airway. However, the source of the mucin has not been determined. So, investigation of the effect of NP or EA on mucin secretion from different kind of secretory cells is needed. Furthermore, the pathways for inhibiting the mucin secretion should be clarified in our next experiments.

The anatomical locations of acupoints for LU1 and CV22 in our quail model are those corresponding to LU1 and CV22 in human thoracic region. To investigate the effect of these human acupoints in our quail model, we made the assumption of that quails are anatomically similar to humans. Thus, we expected that the locations of acupoints in quails are equivalent to those in humans. Much of the research on acupuncture in animals has been conducted based on this assumption, although there is a controversy on whether it is valid to extrapolate the results of acupuncture research from one species to another [23]. Anyway, our experimental results in the quail model have partly provided the clinical empirical effects by acupuncture with scientific evidences. Further studies on patients with the above objective parameters in standard clinical trials are needed. The results from the clinical trials may, in turn, support the assumption of acupoint similarity in different species in this study.

## Conclusion

We conclude that HNE produces a reliable pathogenic model with important features of airway inflammation. This model may be very useful in the development and evaluation of mucoactive therapies. In the present experiments, either EA or NP may act favorably on airway clearance through improving rheological properties of airway secretion and facilitation of MTV. Therefore, our present results seem to be related to the airway clearance action of either EA or NP on obstructive respiratory diseases with airway inflammation in traditional clinical use.

With its special action, either EA or NP shows potential as a useful and popular complementary therapy for the treatment of MC impairment in obstructive respiratory diseases with airway inflammation. The EA and NP, as complementary therapies, may be viewed as acceptable by more and more patients with obstructive respiratory diseases and by the clinicians who treat them. Further rigorous clinical studies to examine their effectiveness in alleviating the symptoms associated with clearance difficulty and viscous secretion retention in the patients need to be carried out.

## List of abbreviation

Chronic obstructive pulmonary diseases (COPD), mucociliary clearance (MC), electro-acupuncture (EA), needle puncture (NP), human neutrophil elastase (HNE), mucociliary transport velocity (MTV), phosphate buffered saline without Ca^2+ ^and Mg^2+ ^(PBS (-))

## Competing interests

The author(s) declare that they have no competing interests.

## Authors' contributions

ST-participated the design of the study, the whole experiment and the drafting of the manuscript

JW-participated the design of the study, the mucociliary transport velocity experiment and the drafting of the manuscript

FS-participated the design of the study, the whole experiment and the drafting of the manuscript

SX-participated the drafting of the manuscript

TW-participated the drafting of the manuscript

MK-supervised the design and the coordination of the study and the drafting of the manuscript

All authors read and approved the final manuscript.

## Pre-publication history

The pre-publication history for this paper can be accessed here:



## References

[B1] Sun G, Sheng C, Yan J (2003). Acupuncture.

[B2] Cheng X, Cheng Y, Huang X, Jia W, Li S, Qiu M, Yang J (1987). Chinese Acupuncture and Moxibustion.

[B3] Li Q, Dong J (2000). Review on the study of the mechanism of acupuncture in the treatment of asthma. Zhongguo Zhong Xi Yi Jie He Za Zhi.

[B4] Maa SH, Sun MF, Hsu KH, Hung TJ, Chen HC, Yu CT, Wang CH, Lin HC (2003). Effect of acupuncture or acupressure on quality of life of patients with chronic obstructive asthma: a pilot study. Journal of Alternative & Complementary Medicine.

[B5] Sternfeld M, Fink A, Bentwich Z, Eliraz A (1989). The role of acupuncture in asthma: changes in airways dynamics and LTC4 induced LAI. Am J Chlin Med.

[B6] Jobst KA (1995). A critical analysis of acupuncture in pulmonary disease: efficacy and safety of the acupuncture needle. Journal of Alternative & Complementary Medicine.

[B7] Blanc PD, Trupin L, Earnest G, Katz PP, Yelin EH, Eisner MD (2001). Alternative therapies among adults with a reported diagnosis of asthma or rhinosinusitis: data from a population-based survey. Chest.

[B8] Cook D, Meade M, Guyatt g, Butler R, Aldawood A, Epstein S (2001). Trials of miscellaneous interventions to wean from mechanical ventilation. Chest.

[B9] Miller AL (2001). The etiologies, pathophysiology, and alternative/complementary treatment of asthma. Alternative Medicine Review.

[B10] National Health Institute (NIH) (1998). NIH Consensus conference. Acupuncture. JAMA.

[B11] Wanner A, Salathe M, O'Riordan TG (1996). Mucociliary clearance in the airways. Am J Respir Crit Care Med.

[B12] Andersson S, Lundebeg T (1995). Acupuncture-from empiricism to science: functional background to acupuncture effects in pain and disease. Medical Hypotheses.

[B13] Xu J (1989). Influence of acupuncture on human nasal mucociliary transport. Chinese Journal of Otorhinolaryngology.

[B14] Mikhireva MM, Portenko GM (1990). Electroacupuncture in combination with surgical intervention in the treatment of patients with polypous rhinosinusitis. Vestnik Otorinolaringologi.

[B15] Tai S, Sun F, O'Brien D, Lee M, Zayas JG, King M (2002). Evaluation of a mucoactive herbal drug, Radix Ophiopogonis, in a pathogenic quail model. Journal of Herbal Pharmacotherapy.

[B16] Tai S, Wang J, Sun F, O'Brien DW, Zayas JG, King M (2003). A pilot study in evaluating the effect of electro-acupuncture on mucociliary clearance in quails [abstract]. Am J Respir Crit Care Med.

[B17] Gibbons MN (1955). The determination of methylpentoses. Analyst.

[B18] Bradford MM (1976). A rapid and sensitive method for the quantitation of microgram quantities of protein utilizing the principle of protein-dye binding. Anal Biochem.

[B19] Tai S, Kai H, Kido T, Isohama Y, Takahama K, Miyata T (1997). Effect of human neutrophil elastase on tracheal mucociliary transport in anesthetized quails. Jpn J Pharmacol.

[B20] Tai S, Kai H, Isohama Y, Moriuchi H, Hagino N, Miyata T (1999). The effect of Maimendongtang on airway clearance and secretion. Phytotherapy Research.

[B21] Tai S, Kai H, Satake Y, Isohama Y, Miyata T (1999). Effect of ophiopogonin on elastase-induced tracheal mucociliary impairment in quail model [abstract]. Am J Respir Crit Care Med.

[B22] Yoshitake K, Kai H, Isohama Y, Hisatsune A, Takahama K, Miyata T (1995). Activated polymorphonuclear leucocytes stimulate the loss of the cell-associated high-molecular weight glycoconjugates from hamster tracheal epithelial cells in culture. Pharmac Sci.

[B23] Schoen AM (2001). Veterinary Acupuncture: ancient art to modern medicine.

